# Supplementary material for: Mechanisms of increased Alzheimer’s disease pathology with R47H and R62H TREM2 variants
Source: Acta Neuropathol. 2026 Jun 15;151(1):67. doi: 10.1007/s00401-026-03036-z (PMC13269326; doi:10.1007/s00401-026-03036-z)
Supplement: Supplementary file 2 — Supplementary file2 (DOCX 3746 KB) [file 401_2026_3036_MOESM2_ESM.docx]

## **Mechanisms of increased Alzheimer’s Disease pathology with R47H and R62H TREM2 variants:** Supplementary Figures

Nurun N. Fancy^1,2^, Nanet Willumsen^1,2^, Vicky MN Chau^1,2^, Samuel L Boulger^1,2^, Harry J. Whitwell^3,4^, Wenhao Wang^3,4^, Baptiste Avot^1,2^, Michael Thomas^1,2^, Jonathan Talbot-Martin^1,2^, Stergios Tsartsalis^5^, Combiz Khozoie^1,2^, Aisling McGarry^1,2,6^, Eleonore Schneegans^1,2^, Riad Yagoubi^1,2^, To Ka Dorcas Cheung^1,2^, Marianna Papageorgopoulou^1,2,^, Emily Adair^1,2^, Benjamin Cooper^4^, Karen Davey^1,2,7^, Amy M Smith^1,2,8^, William Scotton^9,10^, John Hardy^9^, Paul M. Matthews^1,2,11#^, Johanna S. Jackson^1,2#^

Affiliations

^1^UK Dementia Research Institute at Imperial College, UK

^2^Department of Brain Sciences, Imperial College London, UK

^3^Section of Bioanalytical Chemistry, Division of Systems Medicine, Department of Metabolism, Digestion and Reproduction, Sir Alexander Fleming Building, Imperial College London, UK

^4^National Phenome Centre and Imperial Clinical Phenotyping Centre, Department of Metabolism, Digestion and Reproduction, Imperial College London, UK

^5^Department of Psychiatry, University Hospitals of Geneva, Switzerland+ Centre for Psychiatric Neuroscience, Lausanne University Hospitals, Switzerland

^6^Department of Basic and Clinical Neuroscience, Institute of Psychology, Psychiatry and Neuroscience, King’s College London, London, UK

^7^UK Dementia Research Institute at King’s College London, London, UK

^8^Department of Pharmacology and Clinical Pharmacology and Centre for Brain Research, University of Auckland, New Zealand.

^9^Department of Neurodegenerative Disease, University College London (UCL) Institute of Neurology, UK

^10^ Department of Biomedical Sciences, School of Infection, Inflammation and Immunology, College of Medicine and Health, University of Birmingham, Birmingham, UK.

^11^Rosalind Franklin Institute, Harwell Science and Innovation Campus, Didcot, Oxon, United Kingdom

# joint senior authors:

Corresponding authors:

[johanna.jackson@imperial.ac.uk](mailto:johanna.jackson@imperial.ac.uk)

[p.matthews@imperial.ac.uk](mailto:p.matthews@imperial.ac.uk)


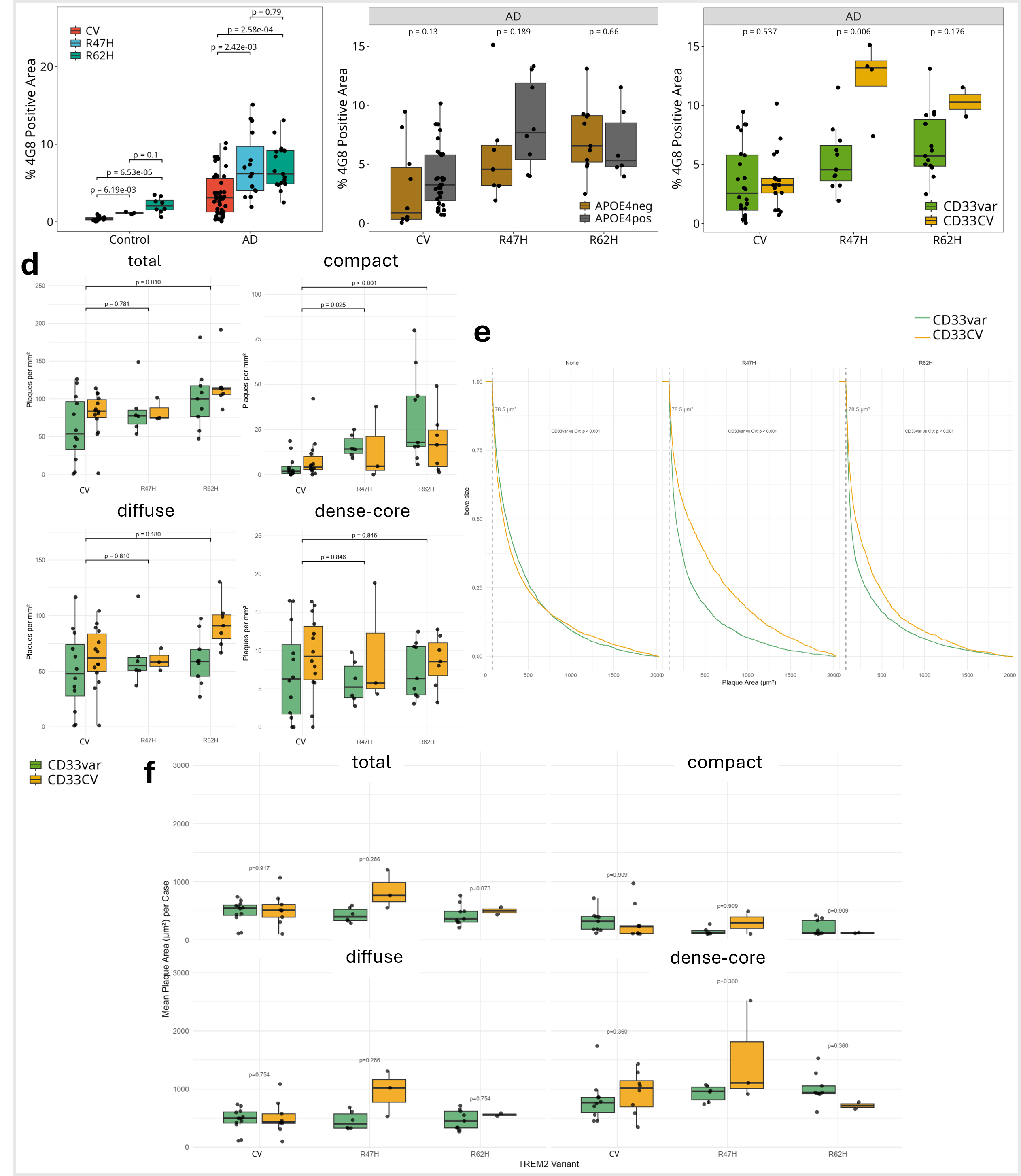


***Supplementary Figure 1*** *Increased amyloid pathology in TREM2var cases.* ***a*** *TREM2var cases had an increase in total amyloid load compared to CV cases.* ***b*** *No statistically significant increase was seen in any TREM2 genotypes in the presence of APOE4 in AD cases.* ***c*** *Increased β-amyloid with TREM2var in MTG and SOM of AD cases, particularly R47H, was partially rescued by the CD33 protective variant (CD33var) compared to the CD33 CV group.* ***d*** *Quantitative IHC of β-amyloid plaques for AD samples in MTG revealed higher total and compact plaque density in R62H cases with no CD33var effect. Statistics are between TREM2 genotypes with no differences between CD33 genotypes.* ***e*** *There was a significant reduction in plaque size with CD33var across all TREM2 genotypes, with the greatest effect seen in R47H cases (Kolmogorov-Smirnov D statistic: TREM2 CV = 0.038, R47H = 0.199, R62H = 0.123).* ***f*** *Plaque size analysis showed a trend towards CD33var reduction of plaque size in R47H cases.*


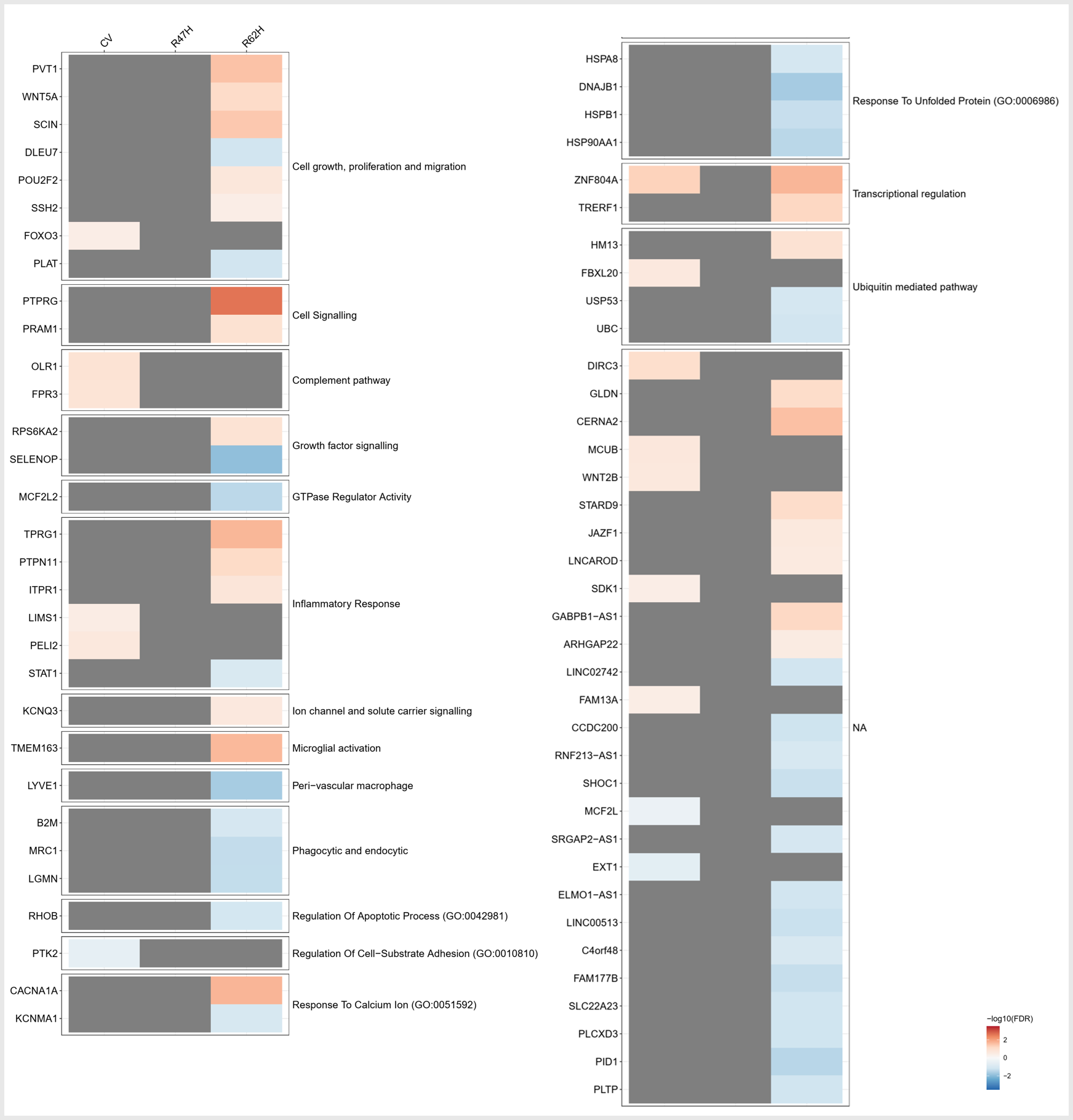


***Supplementary Figure 2*** *TREM2 signature rescued by protective CD33 variant.* *DEG analysis of CD33-TREM2 interaction identified CD33-dependent genes in CV and R62H cases. Most CD33-dependent genes were present within the R62H TREM2var.*


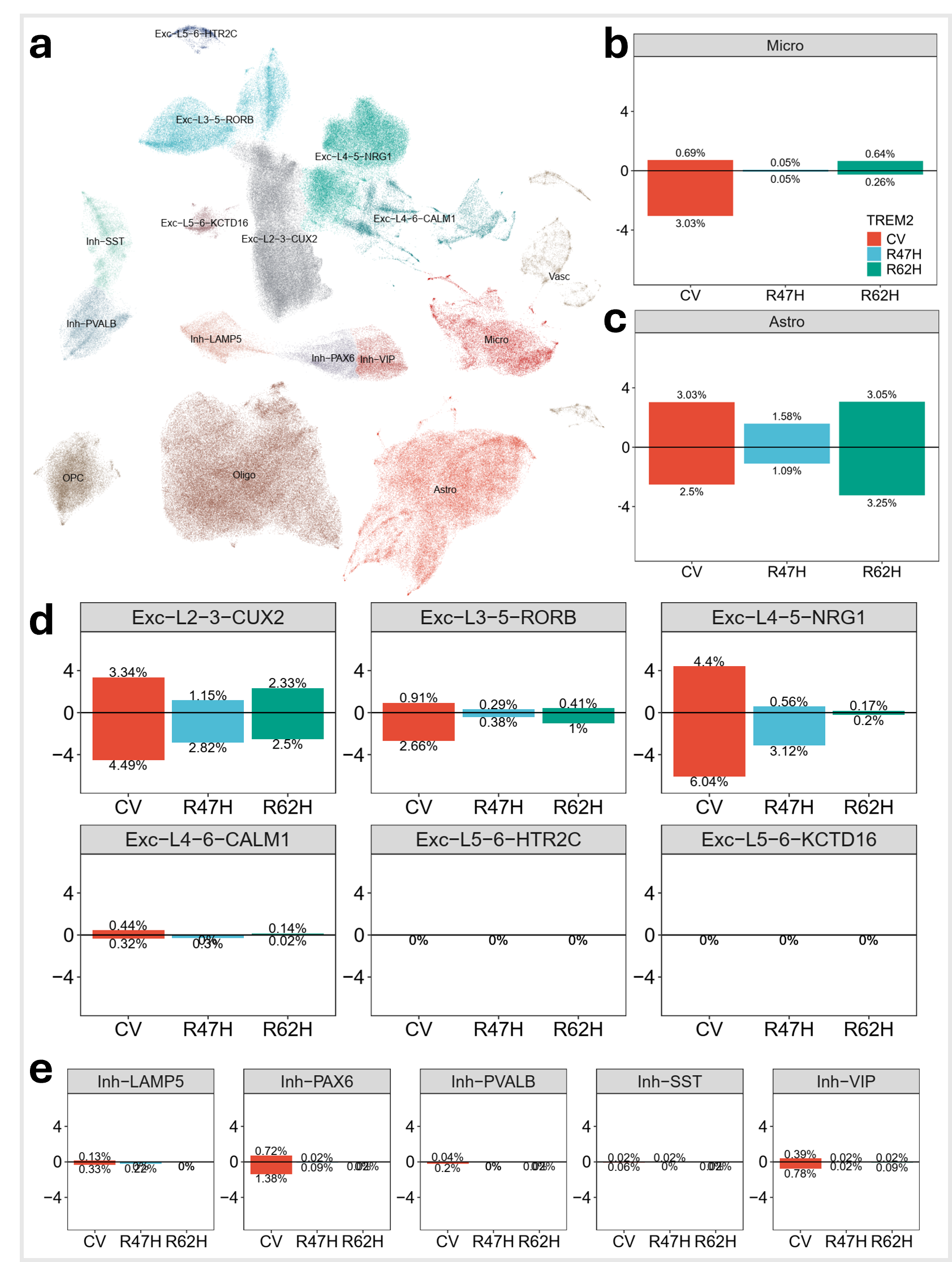


***Supplementary Figure 3*** *Altered differential gene expression (DGE) across TREM2var.* ***a*** *Clusters identified by snRNAseq.* ***b*** *Reduced DGE in R47H microglia.* ***c*** *Reduced DGE in R47H astrocytes.* ***d*** *A broad reduction in DGE across TREM2var excitatory neuronal subtypes.* ***e*** *Reduced DGE in TREM2var inhibitory neuronal subtypes. Note % DGE is based on padj < 0.05 and does not consider fold change.*


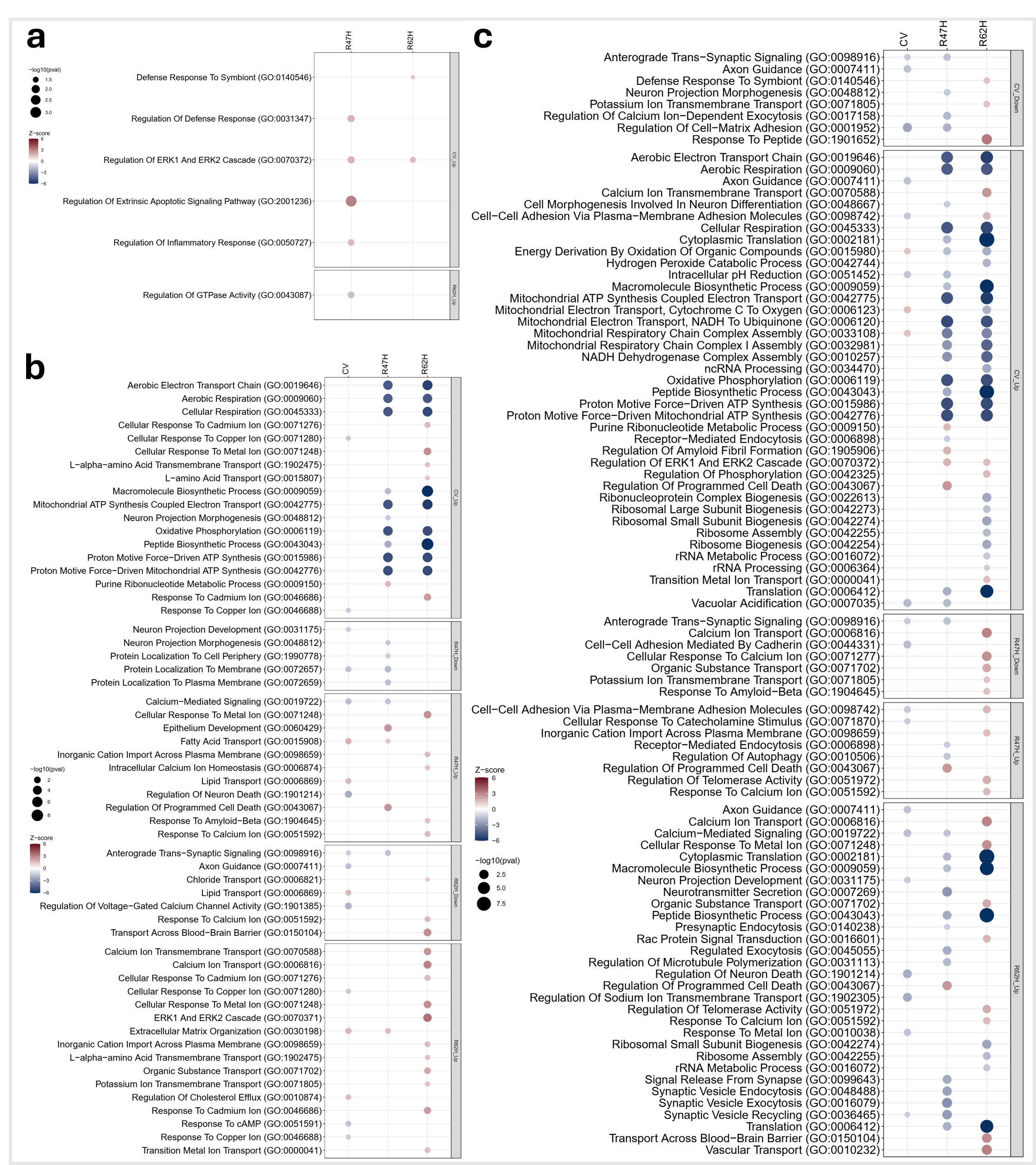


***Supplementary Figure 4*** *Concordant transcriptomic-proteomic pathways across TREM2var cell types.* ***a*** *Few pathways were present at the transcript and protein level in microglia and only in CV cases.* ***b*** *CV astrocytes exhibited the p pathways between RNA and protein compared to TREM2var astrocytes.* ***c*** *R62H* *upregulated transcriptomic pathways had the greatest concordance at the protein level.*
